# An updated annotated checklist of scale insects (Hemiptera, Sternorrhyncha, Coccomorpha) of Poland

**DOI:** 10.3897/zookeys.918.49126

**Published:** 2020-03-12

**Authors:** Bożena Łagowska, Katarzyna Golan

**Affiliations:** 1 Department of Plant Protection, University of Life Sciences in Lublin, Leszczyńskiego 7, 20-069 Lublin, Poland University of Life Sciences in Lublin Lublin Poland

**Keywords:** Coccoids, native and alien species, validation source

## Abstract

A checklist of scale insects recorded to date in Poland is presented. The data provided here are based on literature records and include the latest taxonomic and nomenclatural changes and updates on Coccomorpha reported in Poland. Changes in comparison with ScaleNet and Fauna Europaea electronic databases are also discussed. A total of 185 species belonging to 98 genera and 16 families are included in the list. Of this group, 47 species are alien introduced species and live only indoors, and one species, *Pulvinaria floccifera* (Westwood), develops both indoors and outdoors.

## Introduction

Scale insects form a relatively small group of insects in the Polish fauna and represent only approximately 0.7% of the 27,000 insect species currently known in Poland. However, a few species are identified as pests of economic importance, and especially, in recent years, the invasion of alien scale insects has been observed in several parts of Poland (Łagowska et al. 2015, 2018; Golan et al. 2017).

Scale insects have been known for centuries in Poland for the carmine dye extracted from the Polish cochineal scale *Porphyrophora polonica* (Linnaeus). The presence of *P. polonica* in Poland was reported for the first time in the 16^th^ century (Miechowita 1521) and information about the harmful scale insects was published in later years by some authors (Trzebiński 1916; Ruszkowski 1925, 1933; Minkiewicz 1926). Advanced studies on the scale insect fauna of Poland were initiated by Kawecki, whose results have been presented in numerous publications from 1935 to 1985. In the same period and later, Koteja and Koteja and Żak-Ogaza, in publications from1964 to 2000, extensively studied the Coccomorpha and greatly contributed to the knowledge of the scale insect fauna in Poland. Further contributions are due to the studies by Komosińska (1961-1987), Komosińska and Podsiadło (1967), Dziedzicka (1970-1990), Podsiadło (1975); Podsiadło and Komosińska (1976), Łagowska (1990-2005), Łagowska and Koteja (1996), Dziedzicka and Karnkowski (1999), Łagowska and Golan (2005) and Łagowska et al. (2015, 2017, 2018).

Finally, 90 native and greenhouse species of scale insects that were new to the Polish fauna were discovered in the years 1961–1980 (Koteja 1985; Łagowska and Golan 2005). However, the records of species new to the Polish fauna significantly decreased from 32 in the period 1971–1980 to 8 in 1991–2005 (Łagowska and Golan 2005), and only another 8 new species were recorded in Poland in 2006-2019.

The early data on the distribution of scale insects in Poland were summarized by Kawecki (1985) in a catalogue listing 170 species, including 34 indoor species, and 11 records of misidentified species or species for which no host plants or localities were given. Later, two checklists of scale insects in Poland were presented by Koteja (1996) and Łagowska (2004) who reported 184 and 185 species respectively, each including 44 indoor species. In addition, an annotated list of alien scale insects present in Poland was published by Łagowska et al. (2015).

Two electronic databases provide important world-wide information on scale insect distribution: the Fauna Europaea (FaEu) database (Burckhardt 2013), which reports 163 species of scale insects from Poland, and the ScaleNet database (García Morales et al. 2016), which lists 177 species. Since the last checklist (Łagowska 2004), several new records of scale insects from Poland have been published (Łabanowski 2009; Kalandyk and Węgierek 2010; Kozár et al. 2013; Łagowska et al. 2015, 2018). In the meantime, the nomenclature of the scale insects has also been partially changed. Moreover, several records reported in FaEu and ScaleNet databases were regarded as doubtful or erroneous and need revision. The present paper provides a comprehensive revised list of the scale insects of Poland with updated nomenclature and references to the first reliable Polish records of each species. In addition, discrepancies between the present list and the last checklist (Łagowska 2004) as well as differences from the records reported in the FaEu and ScaleNet databases are discussed.

The aim of the present checklist is to provide baseline reliable data for future faunistic and taxonomic studies.

## Materials and methods

The list presented in this paper is based on the literature records of Coccomorpha in Poland available up to September 2019. A reference to the first reliable record of each species is included. Fossil species of scale insects and those that have been intercepted only once on imported plant materials are excluded. Families and species within each family are listed in alphabetical order according to the classification used in the ScaleNet database (García Morales et al. 2016). The references to species recorded in Poland reported in FaEu and ScaleNet have been checked and, if erroneous, corrected in the present lists. Changes in systematic status and synonymies, mostly proposed by Kozár et al. (2013) and Danzig and Gavrilov-Zimin (2014, 2015), and presently accepted in ScaleNet database, have been adopted in the present list. Scale insect species recorded in Poland are listed in Table 1. They belong to four categories as follows: (i) native species; (ii) alien species established outdoors; (iii) alien species established indoors; and (iv) alien species that can live and develop both outdoors and indoors. The definition of alien species in this paper is the one proposed by Łagowska et al. (2015).

## Results

At the present time the Polish scale insect fauna comprises a total of 185 species, distributed in 98 genera and 16 families. The Pseudococcidae are the most numerous family, with 50 recorded species, followed by Diaspididae (48 species), Coccidae (43 species), and Eriococcidae (sensu lato) (18 species) (Table 1). The remaining 12 families are each represented by 1–5 species. The ratio of species to genera differs between families. The highest ratio (2.6:1) is in the Eriococcidae, followed by Coccidae (2.3:1), Diaspididae (2.0:1), and Kermesidae (2.0:1) (Table 2). The ratio of species number per genus in the Pseudococcidae family is 1.8:1, which is close to the general mean ratio of 1.9:1 reported for Poland (Table 2).

**Figure 1.**
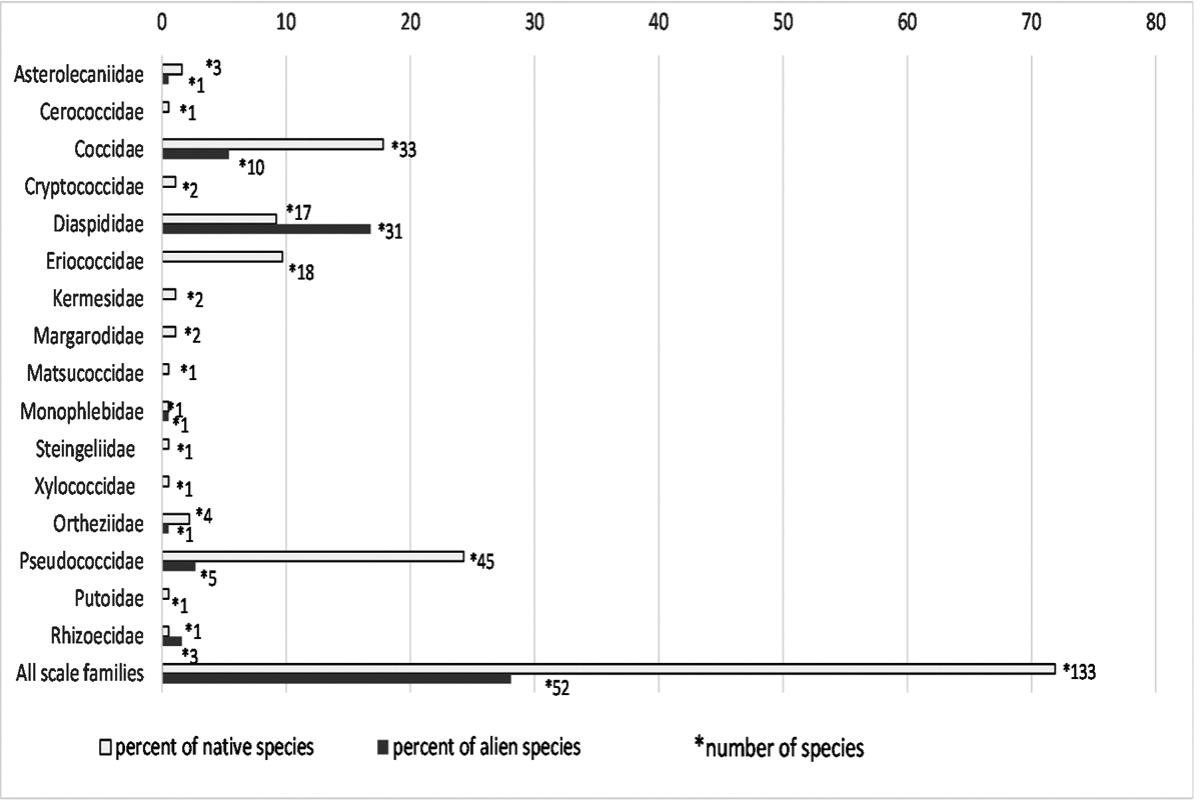
Native and alien scale insect species in different families in Poland.

Of the 185 species present in Poland, 133 (71.9%) are native (Figure 1). The alien scale insect species number 52 (28.1% of total); these species clearly dominate over the native ones in the Diaspididae family, while the remaining families are represented by 1–10 alien species or only by native species (Figure 1). Of the 52 alien species known in Poland, 47 can develop only indoors, while five, namely *Aulacaspis rosae*, *Comstockaspis perniciosa*, *Parthenolecanium fletcheri*, *Pulvinaria floccifera*, and *P. hydrangeae*, overwinter and develop outdoors. *Pulvinaria floccifera* develops both indoors and outdoors (Table 1).

**Table 1.** Checklist of scale insects (Hemiptera: Sternorrhyncha: Coccomorpha) of Poland (* alien established indoors only; ** alien established outdoors; ***alien established indoors and outdoors.

Taxa	Validation source
** Asterolecaniidae **	
1. *Asterodiaspis quercicola* (Bouché, 1851)	Boratyński 1961
2. *Asterodiaspis variolosa* (Ratzeburg, 1870)	Wünn 1919
3. *Asterolecanium epidendri* (Bouché, 1844)*	Kawecki 1985
4. *Planchonia arabidis* Signoret, 1876	Komosińska and Podsiadło 1967
** Cerococcidae **	
5. *Antecerococcus intermedius* (Balachowsky, 1930)	Koteja 1984
** Coccidae **	
6. *Ceroplastes rusci* (Linnaeus, 1758)*	Szulczewski 1926
7. *Coccus hesperidum* Linnaeus, 1758*	Brischke 1883
8. *Eriopeltis festucae* (Fonscolombe, 1834)	Szulczewski 1921
9. *Eriopeltis lichtensteini* Signoret, 1877	Szulczewski 1921
10. *Eriopeltis stammeri* Schmutterer, 1952	Komosińska and Podsiadło 1967
11. *Eucalymnatus tessellatus* (Signoret, 1873)*	Koteja 1972
12. *Eulecanium ciliatum* (Douglas, 1891)	Wünn 1919
13. *Eulecanium douglasi* (Šulc, 1895)	Żak-Ogaza 1961
14. *Eulecanium franconicum* (Lindinger, 1912)	Kawecki 1938
15. *Eulecanium sericeum* (Lindinger, 1906)	Kawecki 1938
16. *Eulecanium tiliae* (Linnaeus, 1758)	Kawecki 1935
17. *Lecanopsis formicarum* Newstead, 1893	Koteja 1969
18. *Lecanopsis subterranea* (Gomez-Menor Ortega, 1948)	Koteja and Żak-Ogaza 1969
19. *Luzulaspis dactylis* Green, 1928	Żak-Ogaza and Koteja 1964
20. *Luzulaspis frontalis* Green, 1928	Koteja 1964
21. *Luzulaspis grandis* Borchsenius, 1952	Żak-Ogaza and Koteja 1964
22. *Luzulaspis luzulae* (Dufour, 1864)	Kawecki 1938
23. *Luzulaspis nemorosa* Koteja, 1966	Koteja 1966
24. *Luzulaspis scotica* Green, 1926	Komosińska and Podsiadło 1967
25. *Nemolecanium graniforme* (Wünn, 1921)	Wünn 1919
26. *Palaeolecanium bituberculatum* (Signoret, 1873)	Kawecki 1935
27. *Parafairmairia bipartita* (Signoret, 1872)	Żak-Ogaza and Koteja 1964
28. *Parafairmairia gracilis* Green, 1916	Koteja and Żak-Ogaza 1969
29. *Parasaissetia nigra* (Nietner, 1861)*	Kawecki 1985
30. *Parthenolecanium corni* (Bouché, 1844)	Lindinger 1911
31. *Parthenolecanium fletcheri* (Cockerell, 1893)**	Kawecki 1935
32. *Parthenolecanium perlatum* (Cockerell, 1898)*	Dziedzicka and Madro 1999
33. *Parthenolecanium persicae* (Fabricius, 1776)	Ruszkowski 1922 (recognized as a doubtful species by Kawecki (1985) and confirmed in Poland by Łagowska (2005a))
34. *Parthenolecanium pomeranicum* (Kawecki, 1954)	Kawecki 1954
35. *Parthenolecanium rufulum* (Cockerell, 1903)	Kawecki 1957
36. *Parthenolecanium smreczynskii* (Kawecki, 1967)	Kawecki 1967
37. *Phyllostroma myrtilli* (Kaltenbach, 1874)	Kawecki 1957
38. *Physokermes hemicryphus* (Dalman, 1826)	Wünn 1919
39. *Physokermes piceae* (Schrank, 1801)	Kawecki 1935
40. *Psilococcus ruber* Borchsenius, 1952	Koteja 1969
41. *Pulvinaria floccifera* (Westwood, 1870)***	Koteja 1972
42. *Pulvinaria hydrangeae* Steinweden, 1946**	Łagowska (unpublished)
43. *Pulvinaria regalis* Canard, 1968	Łagowska et al. 2018
44. *Pulvinaria vitis* (Linnaeus, 1758)	Szulczewski 1921
45. *Saissetia coffeae* (Walker, 1852)*	Szulczewski 1926
46. *Saissetia oleae* (Olivier, 1791)*	Czyżewski 1937
47. *Sphaerolecanium prunastri* (Fonscolombe, 1834)	Kawecki 1957
48. *Vittacoccus longicornis* (Green, 1916)	Koteja 1969
** Cryptococcidae **	
49. *Cryptococcus fagisuga* Lindinger, 1936	Szulczewski 1921
50. *Pseudochermes fraxini* (Kaltenbach, 1860)	Szulczewski 1926
** Diaspididae **	
51. *Aonidia lauri* (Bouché, 1833)*	Komosińska 1968
52. *Aonidiella aurantii* (Maskell, 1879)*	Dziedzicka 1988b
53. *Aspidiotus destructor* Signoret, 1869*	Karnkowski 1993
54. *Aspidiotus nerii* Bouché, 1833*	Ruszkowski 1933
55. *Aspidiotus palmarum* Bouché, 1834*	Schander 1910
56. *Aulacaspis rosae* (Bouché, 1833)**	Trzebiński 1916
57. *Aulacaspis yasumatsui* Takagi, 1977*	Łabanowski 2009
58. *Carulaspis juniperi* (Bouché, 1851)	Szulczewski 1926
59. *Chionaspis salicis* (Linnaeus, 1758)	Wünn 1919
60. *Chrysomphalus aonidum* (Linnaeus, 1758)*	Czyżewski 1937
61. *Chrysomphalus dictyospermi* (Morgan, 1889)*	Dziedzicka 1989
62. *Comstockaspis perniciosa* (Comstock, 1881)**	Kawecki 1950
63. *Diaspidiotus alni* (Marchal, 1909)	Kawecki 1935 (recognized as a doubtful species by Kawecki (1985) and confirmed in Poland by Łagowska (2002)
64. *Diaspidiotus bavaricus* (Lindinger, 1912)	Kawecki 1948
65. *Diaspidiotus gigas* (Thiem & Gerneck, 1934)	Komosińska 1974
66. *Diaspidiotus marani* (Zahradnik, 1952)	Krzysztofowicz 1957
67. *Diaspidiotus ostreaeformis* (Curtis, 1843)	Szulczewski 1921
68. *Diaspidiotus pyri* (Lichtenstein, 1881)	Szulczewski 1921
69. *Diaspidiotus zonatus* (Fauenfeld, 1868)	Szulczewski 1921
70. *Diaspis boisduvalii* Signoret, 1869*	Czyżewski 1975
71. *Diaspis bromeliae* (Kerner, 1778)*	Kawecki 1985
72. *Diaspis echinocacti* (Bouché, 1883)*	Czyżewski 1937
73. *Dynaspidiotus abietis* (Schrank, 1776)	Kawecki 1935
74. *Dynaspidiotus britannicus* (Newstead, 1898)*	Szulczewski 1926
75. *Furchadaspis zamiae* (Morgan, 1890) *	Komosińska 1968
76. *Gymnaspis aechmeae* Newstead, 1898*	Komosińska 1961
77. *Hemiberlesia cyanophylli* (Signoret, 1869) *	Komosińska 1961
78. *Hemiberlesia gliwicensis* (Komosińska, 1965)*	Komosińska 1965
79. *Hemiberlesia lataniae* (Signoret, 1869)*	Czyżewski 1937
80. *Hemiberlesia palmae* (Cockerell, 1892)*	Komosińska 1961
81. *Hemiberlesia rapax* (Comstock, 1881)*	Komosińska 1961
82. *Howardia biclavis* (Comstock, 1883)*	Dziedzicka 1987
83. *Kuwanaspis pseudoleucaspis* (Kuwana, 1902)*	Komosińska 1968
84. *Lepidosaphes conchiformis* (Gmelin, 1790)	Komosińska 1969
85. *Lepidosaphes juniperi* (Lindinger, 1912)	Komosińska 1969
86. *Lepidosaphes newsteadi* (Šulc, 1895)	Komosińska 1974
87. *Lepidosaphes tokionis* (Kuwana, 1902)*	Łabanowski 2009
88. *Lepidosaphes ulmi* (Linnaeus, 1758)	Trzebiński 1916
89. *Leucaspis loewi* Colvée, 1882	Szulczewski 1921
90. *Leucaspis pini* (Hartig, 1839)	Szulczewski 1921
91. *Parlatoria parlatoriae* (Šulc, 1895)*	Żak-Ogaza and Koteja 1964
92. *Parlatoria pergandii* Comstock, 1881*	Komosińska 1964
93. *Parlatoria proteus* (Curtis, 1843)*	Szulczewski 1926
94. *Pinnaspis aspidistrae* (Signoret, 1869)*	Szulczewski 1926
95. *Pinnaspis strachani* (Cooley, 1899)*	Komosińska 1961
96. *Pseudaulacaspis pentagona* (Targioni-Tozzetti, 1886)*	Dziedzicka and Karnkowski 1999
97. *Rhizaspidiotus canariensis* (Lindinger, 1911)	Łagowska 1990
98. *Umbaspis regularis* (Newstead, 1911)*	Komosińska 1968
** Eriococcidae **	
99. *Acanthococcus aceris* Signoret, 1875	Kawecki 1957
100. *Acanthococcus macedoniensis* Fetykó & Kaydan, 2013	Kozár et al. 2013
101. *Anophococcus agropyri* (Borchsenius, 1949)	Koteja, Żak-Ogaza 1966
102. *Anophococcus confusus* (Danzig, 1962)	Koteja 1971a
103. *Anophococcus herbaceus* (Danzig, 1962)	Żak-Ogaza and Koteja 1964
104. *Anophococcus inermis* (Green, 1915)	Żak-Ogaza and Koteja 1964
105. *Anophococcus insignis* (Newstead, 1891)	Żak-Ogaza and Koteja 1964
106. *Anophococcus pseudinsignis* (Green, 1921)	Koteja and Żak-Ogaza 1969
107. *Gossyparia spuria* (Modeer, 1778)	Trzebiński 1916
108. *Greenisca brachypodii* Borchsenius & Danzig, 1966	Koteja and Żak-Ogaza 1966
109. *Greenisca gouxi* (Balachowsky, 1954)	Koteja and Żak-Ogaza 1983
110. *Kaweckia glyceriae* (Green, 1921)	Żak-Ogaza and Koteja 1964
111. *Neokaweckia laeticoris* (Tereznikova, 1965)	Koteja and Żak-Ogaza 1989
112. *Rhizococcus cantium* (Williams, 1985)	Łagowska and Koteja 1996
113. *Rhizococcus devoniensis* Green,1896	Koteja and Żak-Ogaza 1979
114. *Rhizococcus greeni* (Newstead, 1898)	Żak-Ogaza and Koteja 1964
115. *Rhizococcus munroi* (Boratyński, 1962)	Komosińska and Podsiadło 1967
116. *Rhizococcus palustris* Dziedzicka & Koteja, 1971	Dziedzicka and Koteja 1971
** Kermesidae **	
117. *Kermes quercus* (Linnaeus, 1758)	Szulczewski 1921
118. *Kermes* roboris (Fourcroy,1785)	Koteja and Żak-Ogaza 1983
** Margarodidae **	
119. *Neomargarodes festucae* Archangelskaja, 1935	Jakubski 1965
120. *Porphyrophora polonica* (Linnaeus, 1758)	Miechowita 1521
** Matsucoccidae **	
121. *Matsucoccus pini* (Green, 1925)	Boratyński 1960
** Monophlebidae **	
122. *Icerya purchasi* Maskell, 1879*	Chałańska and Łabanowski 2002
123. *Palaeococcus fuscipennis* (Burmeister, 1835)	Szulczewski 1921
** Steingeliidae **	
124. *Steingelia gorodetskia* Nasonov, 1908	Nasonov 1908
** Xylococcidae **	
125. *Xylococcus filiferus* Löw, 1883	Kawecki 1948
** Ortheziidae **	
126. *Arctorthezia cataphracta* (Olafson, 1772)	Kawecki 1938
127. *Newsteadia floccosa* (De Geer, 1778)	Kawecki 1938
128. *Insignorthezia insignis* (Browne, 1887)*	Ruszkowski 1933
129. *Orthezia urticae* (Linnaeus, 1758)	Nowicki 1868
130. *Ortheziola vejdovskyi* Šulc, 1895	Komosińska and Podsiadło 1967
** Pseudococcidae **	
131. *Atrococcus cracens* Williams, 1962	Koteja 1971a
132. *Atrococcus paludinus* (Green, 1921)	Koteja 1971a
133. *Balanococcus boratynskii* Williams, 1962	Koteja 1986
134. *Boreococcus ingricus* Danzig, 1960	Koteja 1986
135. *Brevennia pulveraria* (Newstead, 1892)	Żak-Ogaza and Koteja 1964
136. *Ceroputo pilosellae* Šulc, 1898	Kawecki 1948
137. *Chaetococcus sulcii* (Green, 1934)	Koteja and Żak-Ogaza 1969
138. *Coccura comari* (Künow, 1880)	Koteja et al. 1978
139. *Dysmicoccus angustifrons* (Hall, 1926)	Koteja and Żak-Ogaza1979
140. *Dysmicoccus walkeri* (Newstead, 1891)	Komosińska and Podsiadło 1967
141. *Fonscolombia abdita* (Borchsenius, 1949)	Koteja 1971a
142. *Fonscolombia europaea* (Newstead, 1897)	Koteja and Żak-Ogaza 1969
143. *Fonscolombia tomlinii* (Newstead, 1892)	Koteja 1972
144. *Heliococcus bohemicus* Šulc, 1912	Komosińska 1977
145. *Heliococcus destructor* Borchsenius, 1941	Koteja et al. 1978
146. *Heliococcus sulcii* Goux, 1934	Łagowska and Koteja 1996
147. *Heterococcus nudus* (Green, 1926)	Żak-Ogaza and Koteja 1964
148. *Kiritshenkella lianae* Koteja, 1988	Koteja 1988
149. *Metadenopus festucae* Šulc, 1933	Koteja and Żak-Ogaza 1969
150. *Mirococcopsis subterranea* (Newstead, 1893)	Koteja and Żak-Ogaza1969
151. *Mirococcus clarus* Borchsenius, 1949	Koteja 1971b
152. *Mirococcus festucae* Koteja, 1971	Koteja 1971b
153. *Nipaecoccus nipae* (Maskell, 1893)*	Czyżewski 1937
154. *Peliococcopsis parviceraria* (Goux, 1937)	Koteja et al. 1978
155. *Peliococcus calluneti* (Lindinger, 1912)	Koteja and Żak-Ogaza1966
156. *Peliococcus morrisoni* (Kiritchenko, 1936)	Łagowska 2005b
157. *Pelionella balteata* (Green, 1928)	Koteja 1972
158. *Pelionella manifecta* (Borchsenius, 1949)	Koteja and Żak-Ogaza1989
159. *Phenacoccus aceris* (Signoret, 1875)	Wünn 1919
160. *Phenacoccus hordei* (Lindeman, 1886)	Koteja and Żak-Ogaza 1979
161. *Phenacoccus interruptus* Green, 1923	Żak-Ogaza and Koteja 1964
162. *Phenacoccus phenacoccoides* (Kiritchenko, 1932)	Łagowska and Koteja 1996
163. *Phenacoccus piceae* (Löw, 1883)	Kawecki 1935
164. *Planococcus citri* (Risso, 1813)*	Szulczewski 1926
165. *Planococcus vovae* (Nasonov, 1908)	Kawecki 1948
166. *Pseudococcus longispinus* (Targioni Tozzetti, 1867)*	Szulczewski 1926
167. *Pseudococcus maritimus* (Ehrhorn, 1900)*	Dziedzicka 1988a
168. *Rhodania occulta* Schmutterer, 1952	Koteja and Żak-Ogaza1966
169. *Rhodania porifera* Goux, 1935	Koteja and Żak-Ogaza1969
170. *Saccharicoccus isfarensis* (Borchsenius, 1949)	Koteja 1969
171. *Spilococcus mamillariae* (Bouchѐ, 1844)*	Łabanowski 2009
172. *Trionymus aberrans* Goux, 1938	Koteja and Żak-Ogaza 1966
173. *Trionymus hamberdi* (Borchsenius, 1949)	Danzig 1985 (confirmed in Poland by Łagowska and Koteja (1996))
174. *Trionymus newsteadi* (Green, 1917)	Koteja and Żak-Ogaza1966
175. *Trionymus perrisii* (Signoret, 1875)	Koteja 1969
176. *Trionymus phalaridis* Green, 1925n	Komosińska 1980
177. *Trionymus placatus* (Borchsenius, 1949)	Koteja and Łagowska 1986
178. *Trionymus radicum* (Newstead, 1895)	Koteja 1971a
179. *Trionymus thulensis* Green, 1931	Koteja 1969
180. *Volvicoccus volvifer* Goux, 1945	Kalandyk and Węgorek 2010
** Putoidae **	
181. *Puto superbus* (Leonardi, 1907)	Łagowska 2000
Rhizoecidae	
182. *Rhizoecus americanus* (Hambleton, 1946)*	Łabanowski 2009
183. *Rhizoecus cacticans* (Hambleton, 1946)*	Kawecki 1985
184. *Rhizoecus dianthi* Green, 1926*	Dziedzicka and Madro 1999
185. *Ripersiella halophila* (Hardy, 1868)	Koteja 1972

**Table 2.** Number of scale insect species per genus in relation to families in Poland.

**Family**	**Number of genus**	**Number of species**	**Ratio of species to genera**
Asterolecaniidae	3	4	1.3:3
Cerococcidae	1	1	1:1
Coccidae	19	43	2.3:1
Cryptococcidae	2	2	1:1
Diaspididae	23	48	2.0:1
Eriococcidae	7	18	2.6:1
Kermesidae	1	2	2.0:1
Margarodidae	2	2	1:1
Matsucoccidae	1	1	1:1
Monophlebidae	2	2	1:1
Steingeliidae	1	1	1:1
Xylococcidae	1	1	1:1
Ortheziidae	5	5	1:1
Pseudococcidae	27	50	1.8:1
Putoidae	1	1	1:1
Rhizoecidae	2	4	2.0:1
All scale families	98	185	1.9:1

## Discussion

The scale insect species recorded in Poland represent only ca. 7.3% of the 2536 species known in the Palearctic region (García Morales et al. 2016) and ca. 41.1% of the 450 species reported in Europe (Pellizzari and Germain 2010). The previous checklist of scale insects of Poland was published 15 years ago (Łagowska 2004) and listed 185 species distributed in nine families and 94 genera. In the present list, the families Cryptococcidae, Matsucoccidae, Monophlebidae, Steingeliidae, Xylococcidae, Putoidae, and Rhizoecidae have been added, using the currently accepted classification of Coccomorpha. Moreover, eleven species new for the country have been added and ten species removed. The new entries are: *Asterodiaspis quercicola*, *Pulvinaria hydrangeae*, *P. regalis*, *Aspidiotus palmarum*, *Aulacaspis yasumatsui*, *Lepidosaphes tokionis*, *Acanthococcus macedoniensis*, *Icerya purchasi*, *Spilococcus mamillariae*, *Volvicoccus volvifer*, and *Rhizoecus americanus*. Of these *V. volvifer*, *P. hydrangeae*, *P. regalis*, and *A. macedoniensis* are established outdoors (Kalandyk and Węgierek 2010; Kozár et al. 2013; Łagowska et al. 2018), whereas *A. yasumatsui*, *L. tokionis*, *S. mamillariae*, and *R. americanus*, are indoors species (Łabanowski 2009). *Icerya purchasi* and *A. palmarum* were overlooked in the previous checklist and are therefore added to the present one. *Asterodiaspis quercicola* is here considered as a valid species (García Morales et al. 2016), despite the fact that Podsiadło (1990) and Stumpf and Lambdin (2006) considered *A. quercicola* and *A. variolosa* as synonyms.

Of the ten species removed from the list, six have been synonymized with other species (*Eulecanium slavum* (Kawecki, 1961), *Lepidosaphes oleae* Leonardi, 1908, *Heliococcus danzigae* Bazarov, 1974, *Trionymus isfarensis* Borchsenius, 1949, *T. singularis* Schmutterer, 1952, and *Phenacoccus evelinae* Tereznikova, 1975). The presence in Poland of the other four species removed from the list, *Ripersia corynephori* Signoret, 1875, *Carulaspis visci* (Schrank, 1781), *Fiorinia fioriniae* (Targioni-Tozzetti, 1867) and *Oceanaspidiotus spinosus* (Comstock, 1883) is here considered as doubtful or erroneous. Kiritchenko (1940) listed *R. corynephori* as found near Warsaw, but Kawecki (1985) assumed that this record was incorrect and the species might be a misidentification of *Pseudococcus parvus* Borchsenius, 1949 (now *Mirococcopsis subterranea* (Newstead, 1893)). Similarly, the record of *C. visci* was very likely incorrect and the species may be a misidentification of *Carulaspis juniperi* (Bouchѐ, 1851). In addition, the records of *F. fioriniae* and *O. spinosus* are questionable because no host plants or localities were given in the list published by Czyżewski (1937). All the species mentioned above have not been collected again since they were first recorded.

Based on the distribution data reported by Łagowska (2001), the native species currently known from Poland are all Palearctic. Almost half of them are widely distributed in this region, and relatively few are known only from three or four countries. This latter group includes the following species: *Anophococcus confusus*, *Rhizococcus cantium*, *Boreococcus ingricus*, *Mirococcus festucae*, and *A. macedoniensis*. Two species, *Parthenolecanium smreczynskii* and *Kiritshenkella lianae*, are known so far only from Poland and are possibly endemic.

Recently, much attention has been paid to the alien species of scale insects that have been introduced or have spread into Poland. This group includes 47 indoor and five outdoor species. One species (*P. floccifera*) has been recorded on ornamental plants in greenhouses in Poland as well as on outdoor ornamentals, mostly on *Ilex* sp., and appears to be established (Łagowska et al. 2017). Of the 47 species established indoors, 29 (61.7%) belong to the Diaspididae. This high proportion of species from Diaspididae family introduced into Poland is similar to the results presented by Pellizzari and Germain (2010) for Europe. According to these authors, the 60 alien species belonging to the Diaspididae account for nearly half (44.6%) of the 130 alien species estimated to occur in Europe. Of the five alien species established outdoors in Poland, only *C. perniciosa* and *P. floccifera* are considered as invasive (Łagowska et al. 2017, 2018). Currently, *C. perniciosa* poses the greatest threat, affecting a number of fruit trees in Poland (Golan et al. 2017).

Some differences in the species richness were found between the data reported in the databases ScaleNet and FaEu and the present checklist. Scale insects that are erroneously recorded as present in Poland in the above recorded databases are discussed below:

### *Antecerococcus cistarum* (Balachowsky, 1927), *A. laniger* (Goux, 1932), and *A. pocilliferus* (Neves, 1954)

The presence of these three species in Poland, cited by the ScaleNet database, is a misunderstanding of the text of Koteja (1984), which records them as present in several European countries (Portugal, France, Algeria, Cyprus) but not in Poland. These three species were not listed by any of the researchers studying the fauna of scale insects in Poland, so they are excluded from the present list. The same species have also been incorrectly recorded in the FaEu database.

### *Asterodiaspis minor* (Russell, 1941)

This species was recorded by Russell (1941) in Poland, but Podsiadło (1975) recognized only *Asterodiaspis quercicola* and *A. variolosa* in Poland based on extensive morphological studies. Since this time, *A. minor* was not listed in the subsequent publications pertaining to the fauna of scale insects in Poland and is excluded from the present list, although it is listed in the ScaleNet database.

### *Epidiaspis leperii* (Signoret, 1869)

In the ScaleNet database Poland is included among the locations of distribution of this species based on the paper of Danzig and Pellizzari (1998). However, the paper does not provide any precise indication of its presence in Poland. The species was also not listed later by the researchers who studied the fauna of scale insects in Poland, so it is excluded from the present list.

### *Kermes bacciformis* Leonardi, 1908 and *Kermes ilicis* (Linnaeus, 1758)

These two species are recorded as hosts of a parasitoid by Sugonyaev (1965), and are cited by the ScaleNet database as records of distribution of scale insects, but this is probably a misunderstanding of the text, as the distribution records concern the parasitoid species and not the scale insects. These two species have also been incorrectly included in the FaEu database.

### *Lecanopsis turcica* (Bodenheimer, 1951)

Poland was included among the countries in which this species is distributed in the FaEu database, but the source of information is missing. As there is no published evidence for the presence of this coccid in Poland, it is excluded from the present list.

### *Leucaspis pusilla* Löw, 1883

This species is erroneously recorded in the ScaleNet catalogue citing Danzig and Pellizzari (1998), but those authors do not mention Poland as a location of its distribution.

### *Matsucoccus matsumurae* (Kuwana, 1905)

Poland was included among the countries where this species is present in the FaEu database, but the source of information is missing. As there is no evidence for the presence of this species in Poland, it is excluded from the present list.

### *Parlatoria oleae* (Colvee, 1880) and *Parlatoria theae* Cockerell, 1896

These two species are recorded as present in Poland in the FaEu database with an incorrect citation of ScaleNet as the source of information. We have been unable to trace the original sources of publication concerning the presence of these species in Poland and therefore we consider these records erroneous.

### *Parlatoria ziziphi* (Lucas, 1853)

This species is cited in ScaleNet based on Komosińska (1964). However, this species was only found on citrus fruits imported to Poland. Since *P. ziziphi* was not mentioned in subsequent papers, we assume that this species is not established in Poland.

### *Pseudococcus viburni* (Signoret, 1875)

Poland was included among the countries in which this species is present in the FaEu database, but the source of information is missing. As there is no evidence of the presence of this mealybug in Poland, this species is excluded from the present list.

### *Trionymus levis* (Tang, 1992)

Koteja (1974) and Koteja and Żak-Ogaza (1983) do not provide evidence of the presence of *T. levis* in Poland as cited by the ScaleNet database. Moreover, this species was not listed in the subsequent publications related to the Polish fauna of scale insects and is therefore removed from the present checklist. It has also been incorrectly included in the FaEu database.

## References

[B1] Boratyński K (1960) *Matsucoccus pini* (Green) (Homoptera: Coccoidea, Margarodidae) in Norfolk. Entomologist’s Monthly Magazine 93: 246–251.

[B2] Boratyński K (1961) A note on the species of *Asterolecanium* Targioni-Tozzeti, 1869 (Homoptera, Coccoidea, Asterolecaniidae) on oak in Britain. Proceedings of the Royal Entomological Society of London, Series B: Taxonomy 30: 4–14. 10.1111/j.1365-3113.1961.tb00435.x

[B3] Brischke CGA (1883) Beschreibung der forst-, garten- und landwirthschaftlichen Feinde und Freunde unter den Insekten. Schriften der Naturforschenden Gesellschaft in Danzig 5(4): 97–125.

[B4] Burckhardt D (2013) Fauna Europaea: Coccoidea version 2017.06. https://fauna-eu.org [Accessed on 11.09.2019]

[B5] Chałańska A Łabanowski G (2002) New and less known plant pests in greenhouses – part I. Hasło Ogrodnicze 5: 86–90. [In Polish]

[B6] Czyżewski JA (1937) The importance of plant diseases and pests in floriculture. Wiadomości Ogrodnicze 3: 15–18. [In Polish]

[B7] Czyżewski JA (1975) Plant Diseases and Pests in Floriculture. Warszawa, 666 pp. [In Polish]

[B8] Danzig EM (1985) Contribution to the scale insect fauna (Homoptera, Coccinea) of Teberda State Reserve. Entomologicheskoe Obozrenye 64: 110–123.

[B9] Danzig EM Gavrilov-Zimin IA (2014) Palaearctic mealybugs (Homoptera: Coccinea: Pseudococcidae), Part 1: Subfamily Phenacoccinae. Russian Academy of Sciences, St. Petersburg, 678 pp.

[B10] Danzig EM Gavrilov-Zimin I.A (2015) Palaearctic mealybugs (Homoptera: Coccinea: Pseudococcidae), Part 2: Subfamily Pseudococcinae. Russian Academy of Sciences, St. Petersburg, 619 pp 10.31610/zsr/2015.24.2.236

[B11] Danzig EM Pellizzari G (1998) Diaspididae. In: Kozár F (Ed.) Catalogue of Palaearctic Coccoidea. Hungarian Academy of Sciences, Budapest, 526 pp.

[B12] Dziedzicka A (1970) Materials for the study of scale insects (Coccoidea) occurring on Polish territory. Rocznik Naukowo-Dydaktyczny, WSP w Krakowie 37: 24–27. [In Polish with English summary]

[B13] Dziedzicka A (1987) Notes on the occurrence of rare species of greenhouse armored scale insects (Homoptera, Coccinea, Diaspididae) in Poland. Rocznik Naukowo-Dydaktyczny WSP w Krakowie 111: 143–150. [In Polish with English summary]

[B14] Dziedzicka A (1988a) Glass-house mealybugs (Homoptera, Coccinea, Pseudococcidae). Zeszyty Problemowe Postępów Nauk Rolniczych 333: 87–91. [In Polish]

[B15] Dziedzicka A (1988b) The greenhouses scale insects of Poland. Rocznik Naukowo-Dydaktyczny, WSP w Krakowie 123: 79–91. [In Polish with English summary]

[B16] Dziedzicka A (1989) Scale insects (Coccinea) occurring in Polish greenhouses. Part I. Diaspididae. Acta Biologica Cracoviensia, Series Zoologia 31: 93–114.

[B17] Dziedzicka A (1990a) The characteristic of scale insects (Coccinea) occurring in Polish greenhouses. Part II. Coccidae. Acta Biologica Cracoviensia, Series Zoologia 32: 17–27.

[B18] Dziedzicka A (1990b) The characteristic of scale insects (Coccinea) occurring in Polish greenhouses. Part III. Pseudococcidae. Acta Biologica Cracoviensia, Series Zoologia 32: 29–38.

[B19] Dziedzicka A Karnkowski W (1999) White peach scale *Pseudaulacaspis pentagona* Targ.-Tozz. – potential threat to orchards and plantations of woody ornamental plants in Poland. Ochrona Roślin 43(8): 30–32. [In Polish]

[B20] Dziedzicka A Koteja J (1971) A revision of the species of the genus *Rhizococcus* Signoret (Homoptera, Coccoidea) occurring in Poland. Acta Zoologica Cracoviensia 16: 557–579.

[B21] Dziedzicka A Madro D (1999) Three species of scale insects (Coccinea) new to Polish greenhouses. Acta Biologica Cracoviensia; Series Zoologia 41: 15–18.

[B22] García Morales M Denno B Miller DR Miller GL Ben-Dov Y Hardy NB (2016) ScaleNet: a literature-based model of scale insect biology and systematics. http://scalenet.info [Accessed on 20.08.2019] 10.1093/database/bav118 PMC4747323 26861659

[B23] Golan K Łagowska B Kot I (2017) *Comstockaspis perniciosa* Comst. (Hemiptera; Coccomorpha; Diaspididae) again in Poland. 35^th^ National Hemipterological Conference, Katowice-Kochcice (Poland), April 2017, 26. [In Polish]

[B24] Jakubski AW (1965) A critical revision of the families Margarodidae and Termitococcidae (Hemiptera, Coccoidea). Trustees of the British Museum (Natural History) London, 187 pp.

[B25] Kalandyk M Węgierek P (2010) Scale Insects (Hemiptera, Sternorrhyncha, Coccoidea) of Selected Plant Communities in the Eastern Part of Garb Tarnogórski. Annals of the Upper Silesian Museum in Bytom – Entomology 19: 1–116.

[B26] Karnkowski W (1993) The occurrence of armored scales *Aspidiotus destructor* Signoret on dracaena plant. Wszechświat 94(5): 127–128. [In Polish]

[B27] Kawecki Z (1935) Scale insects (Coccidae) of the Krakow and Kielce provinces collected in 1933–1934. Sprawozdanie Komisji Fizjograficznej Polskiej Akademii Umiejętności 68/69: 73–90. [In Polish]

[B28] Kawecki Z (1938) The Coccidae of the Tatra Mountains. Sprawozdanie Komisji Fizjograficznej Polskiej Akademii Umiejętności 71: 199–208. [In Polish]

[B29] Kawecki Z (1948) A contribution to the knowledge of scale insects (Coccidae) of Poland. Some Coccidae from Poland. Materiały Fizjograficzne Kraju 10: 1–10. [In Polish]

[B30] Kawecki Z (1950) San José Scale – Quadraspidiotus (Aspidiotus) perniciosus Comst. in Europe and its appearance in Poland. Polska Akademia Umiejętności, Prace Rolniczo-Leśne 55: 1–54. [In Polish]

[B31] Kawecki Z (1954) Studies on the genus *Lecanium* Burm. II. The Yew Scale, *Lecanium pomeranicum* sp. n. and some related species (Homoptera, Coccoidea, Lecaniidae). Annales Zoologici 16: 9–22. [In Polish]

[B32] Kawecki Z (1957) Notes on scale insects (Homoptera, Coccoidea). Acta Zoologica Cracoviensia 2: 193–204. [In Polish]

[B33] Kawecki Z (1967) Studies on the genus *Lecanium* Burm. VI. *Lecanium smreczynskii* sp. n. (Homoptera, Coccoidea, Lecaniidae). Bulletin de l'Academie Polonaise des Sciences 15: 687–689.

[B34] Kawecki Z (1985) Scale insects, Coccoidea. Catalogue of Polish Fauna, Polish Academy, Zoological Institute 21: 1–108. [In Polish].

[B35] Kiritchenko A (1940) Tretie soobščenije o faunie kokcid (Coccoidea). Trudy Zoologicheskogo Instituta. Akademiia Nauk SSSR 6: 115–137.

[B36] Komosińska H (1961) On some scale-insects (Homoptera, Coccoidea) living in greenhouses in Poland. Fragmenta Faunistica 9: 221–232. [In Polish with English summary] 10.3161/00159301FF1961.9.15.221

[B37] Komosińska H (1964) Armored scale insects (Homoptera, Coccoidea, Diaspididae) of citrus fruits imported to Poland. Fragmenta Faunistica 11: 207–231. 10.3161/00159301FF1964.11.14.207

[B38] Komosińska H (1965) A new species of *Abgrallaspis* Balach. (Homoptera, Coccoidea, Diaspididae) from greenhouses in Poland. Frustula Entomologica 8(4): 1–6.

[B39] Komosińska H (1968) Investigations on the scale insects (Homoptera, Coccoidea, Diaspididae) living in greenhouses in Poland. Part I. Polskie Pismo Entomologiczne 38: 205–208. [In Polish with English summary]

[B40] Komosińska H (1969) Studies of scale insects (Homoptera, Coccoidea, Diaspididae) of Poland, I. Fragmenta Faunistica 15: 267–271. [In Polish with English summary] 10.3161/00159301FF1969.15.16.267

[B41] Komosińska H (1974) Physiographical and ecological investigations upon armored scale insects (Homoptera, Doccoidea, Diaspididae) of Poland. Zeszyty Naukowe AR w Warszawie. Rozprawy Naukowe 43: 1–84. [In Polish with English summary]

[B42] Komosińska H (1977) Materials to the knowledge of scale insects (Homoptera, Coccoidea) of the Kampinoski National Park. Sylwan 121(1): 21–24. [In Polish]

[B43] Komosińska H (1980) *Trionymus luzensis* sp. n. (Homoptera, Coccoidea, Pseudococcidae) from Poland. Annales Zoologici (Warsaw) 35: 257–265.

[B44] Komosińska H (1987) Occurrence of scale insects (Homoptera, Coccoidea) on trees and shrubs of the Warsaw parks. Annals of Warsaw Agricultural University – SGGW-AR (21): 95–103.

[B45] Komosińska H Podsiadło E (1967) Materials to the fauna of scale insects (Homoptera, Coccoidea) steppe reservations in the Nida Valley (South Poland). I. Bulletin de l'Academie Polonaise des Sciences. Serie des Sciences Biologiques 15: 683–686.

[B46] Koteja J (1964) Notes on scale insects (Homoptera, Coccoidea) in Poland's fauna. Polskie Pismo Entomologiczne 34: 177–184. [In Polish with English summary]

[B47] Koteja J (1966) *Luzulaspis nemorosa* sp. n. (Homoptera, Coccoidea, Coccidae). Polskie Pismo Entomologiczne 36(3): 45–46.

[B48] Koteja J (1969) Notes on the Poland’s scale insect fauna (Homoptera, Coccoidea). II. Polskie Pismo Entomologiczne 39: 3–15.

[B49] Koteja J (1971a) Notes on the Polish scale insect fauna (Homoptera, Coccoidea). III. Polskie Pismo Entomologiczne 41: 319–326. [In Polish with English summary]

[B50] Koteja J (1971b) Two new species of *Micrococcus* Borchsenius (Homoptera, Coccoidea, Pseudococcidae). Polskie Pismo Entomologiczne 42: 565–571.

[B51] Koteja J (1972) Notes on the Polish scale insect fauna (Homoptera, Coccoidea) IV. Polskie Pismo Entomologiczne 42(3): 565–571.

[B52] Koteja J (1974) The occurrence of a campaniform sensillum on the tarsus in the Coccinea (Homoptera). Polskie Pismo Entomologiczne 44: 243–252.

[B53] Koteja J (1984) *Cerococcus cycliger* (Goux) (Homoptera, Coccinea) new to the Polish Fauna. Polskie Pismo Entomologiczne 54: 413–414.

[B54] Koteja J (1985) Faunistic investigations on scale insects Coccinea (Homoptera, Coccinea) in Poland. Wiadomości Entomologiczne 6: 11–26. [In Polish]

[B55] Koteja J (1986) Notes on the Polish scale insect fauna (Homoptera, Coccinea) VI. Polskie Pismo Entomologiczne 56: 217–219.

[B56] Koteja J (1988) Review of *Kiritshenkella* Borchsenius and *Balanococcus* Williams, with a description of a new species (Homoptera, Pseudococcidae). Annales Zoologici 42: 119–163.

[B57] Koteja J (1996) How to recognize scale insects (Homoptera, Coccinea). In: Boczek J (Ed.) Diagnosis of plant pests and their natural enemies.SGGW Warszawa, 2: 139–231. [In Polish]

[B58] Koteja J (2000) Scale insects (Sternorrhyncha: Coccoidea) from the Bieszczady Mountains. Monografie Bieszczadzkie 7: 241–244.

[B59] Koteja J Łagowska B (1986) *Dysmicoccus balticus* sp. n. (Homoptera, Coccinea, Pseudococcidae). Polskie Pismo Entomologiczne 56: 381–388.

[B60] Koteja J Żak-Ogaza B (1966) Investigations on scale insects (Homoptera, Coccidea) of the Pieniny Klippen Belt. Acta Zoologica Cracoviensia 11: 305–332.

[B61] Koteja J Żak-Ogaza B (1969) The scale insect fauna (Homoptera, Coccidea) of the Ojców National Park in Poland. Acta Zoologica Cracoviensia 14: 351–373.

[B62] Koteja J Żak-Ogaza B (1979) Five species of Pseudococcidae and Eriococcidae (Homoptera) new to the Polish fauna. Polskie Pismo Entomologiczne 49: 671–675.

[B63] Koteja J Żak-Ogaza B (1983) The Coccinea fauna (Homoptera) of the Kraków-Czestochowa Upland (southern Poland). Acta Zoologica Cracoviensia 26: 465–490. [In Polish with English summary]

[B64] Koteja J Żak-Ogaza B (1989) Scale insects (Homoptera: Coccinea) of the Swietokrzyskie Mountains. Fragmenta Faunistica 32: 19–34. [In Polish with English summary] 10.3161/00159301FF1989.32.12.243

[B65] Koteja J Żak-Ogaza B El-Nabawi A (1978) Notes on *Heterococcus* Ferris and four mealybugs (Homoptera, Pseudococcidae) new to the Polish fauna. Polskie Pismo Entomologiczne 48: 501–540.

[B66] Kozár F Kaydan MB Konczné Benedicty Z Szita É (2013) Acanthococcidae and related families of the Palaearctic Region. Hungarian Academy of Sciences, Budapest, Hungary, 680 pp.

[B67] Krzysztofowicz A (1957) A contribution to the knowledge of Polish scale insect fauna (Homoptera, Coccoidea, Aspidiotini). Zeszyty Naukowe Uniwersytetu Jagiellońskiego 10: 223–240. [In Polish]

[B68] Lindinger L (1911) Beiträge zur Kenntnis der Schildläuse und ihre Verbreitung II. Zeitschrift für Wissenschaftliche Insektenbiologie 7: 9–12, 86–90, 126–130, 172–177.

[B69] Łabanowski G (2009) Pests of ornamental plants introduced to Polish glasshouses. Progress in Plant Protection 49(4): 1714–1723.

[B70] Łagowska B (1990) *Rhizaspidiotus canariensis* (Lindinger) (Homoptera, Diaspididae) new to the Polish fauna. Polskie Pismo Entomologiczne 60: 261–263.

[B71] Łagowska B (2000) *Puto superbus* (Leonardi, 1907) (Homoptera: Pseudococcidae) new to the Polish fauna. Polskie Pismo Entomologiczne 69: 3–6.

[B72] Łagowska B (2001) Zoogeographical analysis of the scale insect fauna of Poland. Bollettino di Zoologica Agraria e di Bachicoltura Ser. II, 3(3): 239–248.

[B73] Łagowska B (2002) New data on the occurrence of *Diaspidiotus alni* (Marchal, 1909) (Hemiptera: Coccoidea: Diaspididae) in Poland. Wiadomości Entomologiczne 20(3-4): 184. [In Polish]

[B74] Łagowska B (2004) Species checklist. Sternorrhyncha, scale insects (Coccoidea). In: Bogdanowicz W Chudzicka E Pilipiuk I Skibińska E (Eds) Fauna of Poland – Characteristics and checklist of species.Tom I . Wyd. Muzeum i Instytut Zoologii PAN, Warszawa, 266–269. [In Polish]

[B75] Łagowska B (2005a) New data on the occurrence and morphological variability of *Parthenolecanium persicae* (Fabricius, 1776) (Hemiptera: Coccidae) in Poland. Wiadomości Entomologiczne 24(1): 5–10. [In Polish with English summary]

[B76] Łagowska B (2005b) *Spinococcus morrisoni* (Kiritchenko, 1936) (Hemiptera: Pseudococcidae) – new to the fauna of Poland. Polskie Pismo Entomologiczne 74: 39–42.

[B77] Łagowska B (unpublished) New record and data on *Pulvinaria* species (Hemiptera, Coccomorpha, Coccidae) from Poland. Polish Journal of Entomology.

[B78] Łagowska B Golan K (2005) The condition of faunistic research on scale insects (Hemiptera, Coccinea) in Poland. Aphids and other Hemipterous insects. Monograph 11. Agricultural University Publisher, Poznań, 107-116.

[B79] Łagowska B Koteja J (1996) Scale insects (Homoptera, Coccinea) of Roztocze. Fragmenta Faunistica 39: 29–42. [In Polish with English summary] 10.3161/00159301FF1996.39.4.029

[B80] Łagowska B Golan K Kot I Kmieć K Górska-Drabik E Goliszek K (2015) Alien and invasive scale insect species in Poland and their threat to native plants. Bulletin of Insectology 68(1): 13–22.

[B81] Łagowska B Golan K Kmieć K Kot I Górska-Drabik E Goliszek K (2017) The phenology of *Pulvinaria floccifera* Westwood (Hemiptera: Coccomorpha: Coccidae), a new invasive pest on ornamentals outdoors in Poland. Turkish Journal of Zoology 41: 113–118.

[B82] Łagowska B Golan K Michalski M (2018) First record of *Pulvinaria regalis* Canard, 1968 (Hemiptera: Coccomorpha: Coccidae) in Poland. Polish Journal of Entomology 87(4): 371–378. 10.2478/pjen-2018-0025

[B83] Miechowita M (1521) Chronica polonorum. Cracoviae, cap. 3: III.

[B84] Minkiewicz S (1926) Checklist of major pests on crops in Poland. 2: 1–9. [In Polish]

[B85] Nasonov NV (1908) *Steingelia gorodetskia*, nov. gen. et nov. sp. nouveau genre et espèce des coccides du groupe Xylococcini. [New genus and species of coccids from the group Xylococcini.]. Annuaire du Musée Zoologique de l'Académie Impériale des Sciences de St. Pétersbourg 13: 345–352.

[B86] Nowicki M (1868) Checklist of bugs (Rhynchota F., Hemiptera L.). Sprawozdania Komisji Fizyograficznej Kraków 2: 91–107.

[B87] Pellizzari G Germain JF (2010) Scales (Hemiptera, Superfamily Coccoidea. In: Roques A Kenis M Leeds D Lopez-Vaamonde C Rabitsch W Rasplus JY Roy DB (Eds) Alien terrestrial arthropods of Europe.Pensoft Publishers, Sofia, Bulgaria. BioRisks 4(1), 475–510. 10.3897/biorisk.4.45

[B88] Podsiadło E (1975) Studies on the identification and occurrence of species of the genus asterodiaspis Signoret, 1876 (Homoptera, Coccoidea, Asterolecaniidae). Przegląd Zoologiczny 19(2): 211–216. [In Polish with English summary]

[B89] Podsiadło E (1990) Concept of the species of *Asterodiaspis variolosa* (Ratzeburg, 1870) (Homoptera, Coccoidea, Asterolecaniidae). Annales Zoologici 43(18): 363–371.

[B90] Podsiadło E Komosińska H (1976) Further investigations on the scale insect fauna (Homoptera, Coccoidea) in the Nida Valley (Southern Poland). Bulletin de l'Academie Polonaise des Sciences. Serie des Sciences Biologiques 24: 87–91.

[B91] Russell LM (1941) A classification of the scale insect genus *Asterolecanium*. United States Department of Agriculture, Miscellaneous Publications 424: 1–319. 10.5962/bhl.title.65621

[B92] Ruszkowski J W (1922) Animal pests of fruit orchards around Poznań observed in 1921. Ziemianin, Poznań 73: 264–270. [In Polish]

[B93] Ruszkowski JW (1925) Pests in orchards around Poznań in 1922. Choroby i Szkodniki Roślin 1(1): 32–36. [In Polish]

[B94] Ruszkowski JW (1933) Research results on harmful Polish fauna based on materials from 1919-1930. Roczniki Ochrony Roślin 1(1–3): 1–567. [In Polish]

[B95] Schander R (1910) Bericht über das Auftreten von Krankheiten und tierischen Schädlingen an Kulturpflanzen in den Provinzen Posen und Westpreußen für das Jahr 1908. Mitt. K. Wilh. Inst. Landw. Bromberg 2: 148.

[B96] Stumpf CF Lambdin PL (2006) Pit scales (Sternorrhyncha: Coccoidea) of North and South America. Tennessee Agricultural Experiment Station, University of Tennessee, Institute of Agriculture Knoxville, Tennessee, 231 pp.

[B97] Sugonyaev ES (1965) Palearctic species of the genus *Blastothrix* Mayr (Hymenoptera, Chalcidoidea) with remarks on their biology and useful role. Part 2. Entomological Review 44: 225–233.

[B98] Szulczewski JW (1921) A contribution to fauna Coccidae of Wielkopolska. Prace Komisji Fizjograficznej, Poznań 1: 78–84. [In Polish]

[B99] Szulczewski JW (1926) Data for the scale insect fauna of Poznań. Polskie Pismo Entomologiczne 5: 137–143. [In Polish]

[B100] Trzebiński J (1916) Diseases and pests of plants in the Kingdom of Poland. According to data from the Plant Protection Station from 1912, 1913 and 1914 with earlier data attached. Pamiętnik Fizjograficzny 23(3): 1–106. [In Polish]

[B101] Wünn H (1919) Über die Cocciden des Urwaldes von Bialovies. Abh. Senckenb. Naturforsch. Ges. 37: 1–21.

[B102] Żak-Ogaza B (1961) Research on chalcid wasps (Hymenoptera, Chalcidoidea) parasitoids of scale insects (Homoptera, Coccoidea) from Poland. Polskie Pismo Entomologiczne 31: 349–410. [In Polish]

[B103] Żak-Ogaza B Koteja J (1964) Investigations on scale insects (Homoptera, Coccoidea) of the Pieniny Mountains. Acta Zoologica Cracoviensia 9: 417–439.

